# Views and Attitudes of Patients in Mental Facilities Regarding Smoking

**DOI:** 10.5539/gjhs.v8n8p228

**Published:** 2015-12-17

**Authors:** Michael Kourakos, Athina Kalokairinou, Sophia Zyga, Evmorphia Koukia

**Affiliations:** 1 Department of Nursing, National and Kapodistrian University of Athens, Greece; 2 Department of Nursing, University of Peloponnese, Greece

**Keywords:** smoking, smoking habits, attitudes, beliefs, mental patients

## Abstract

**Introduction:**

Smoking rates amongst people with a mental health disorder are significantly higher than in the general population and there is growing evidence to show a strong association between smoking and mental health disorders. The aim of the present study was to investigate views and attitudes of mental patients regarding smoking.

**Material and Methods:**

The sample is composed of 356 patients treated in the Attica Psychiatric Hospital (a.k.a. “Dafni”) as well as in other Units affiliated with the Hospital. The ‘Smoking in psychiatric hospitals-a survey of patients’ views’ questionnaire was used in the form of semi-structured interviews. The PASW 18 (SPSS Inc.) package was used for the statistical analysis and statistical significance was set to p= 0.05.

**Results:**

Overall, 40% of the participants were in-patients, the rest being treated in other settings, the average length of hospital stay was 4.4 years, and the most common diagnosis (61.5.%) was schizophrenia (F20, according to ICD-10), while almost all of the participants (97.5%) were smokers. Most patients (58.8%) said they had had a hard time trying to quit smoking although they had sufficient information and encouragement (≈90%); they also reported that watching the staff smoking did not affect them inasmuch as watching other patients smoking (41 % vs 54.8%). 75.5% of the patients felt that they had no particular difficulty to quit smoking. Men smoked significantly more cigarettes per day compared to females (36.70 vs 30.82, p=0.002). There were no significant differences among males and females regarding previous attempts to quit smoking. Information gathered from such studies should be taken into account when designing systematic smoking management plans in mental institutions.

**Conclusion:**

Although almost all mental patients smoke, they seem to be receptive to quitting smoking, since two thirds of them have already tried to quit, but one-third of the patients find smoking a little or not at all dangerous.

## 1. Introduction

Exact smoking rates in Greece as well as other countries vary according to region and demographics. A recent study that compares smoking rates between 2006 and 2010 (when the new anti-smoking law was enacted) showed reduced smoking rates especially among young adults from 48% to 36% (Filippidis, Vardavas, Loukopoulou, Behrakis, Connolly, & Tountas, 2013).

In regards to the correlation between nicotine addiction and mental disorders, although no causal links have been established, it has been found that nicotine addicts are 2.7-8.1 times more likely to suffer from depression, stress or bipolar disorder, other personality disorder or attention-deficit hyperactivity disorder compared to non-addicted (occasional/social) smokers, non-smokers and ex smokers (American Psychiatric Association, 2013).

A factor that makes it more difficult to assess the patients’ smoking habits is that during their hospital stay the patients may change their smoking habits which, in turn, could alter the manifestation of the mental disorder and its treatment (Olivier, Lubman, & Fraser, 2007). Moreover, the staff often uses cigarettes to achieve desired behaviors, something that can alter smoking-related social interaction (Olivier et al., 2007). Another obstacle regarding smoking cessation in mental patients was the assumption that smoking was a kind of self-administered treatment for some symptoms, an assumption that kept several doctors from encouraging their patients to quit smoking (Ziedonis et al., 2008). Doubtlessly, for many years there had been a tolerance-even encouragement- for smoking within the field of mental health (Schroeder, & Morris, 2010).

In Great Britain, a study regarding mental patients’ views about smoking found that 76% of the sample (n= 102) were smokers, and 74% of them thought it was too hard for them to quit, although they were fully informed and encouraged by the staff (Dickens, Stubbs, Popham, & Haw, 2005). The majority wanted both visitors and staff to be able to smoke along, and also to be permitted to smoke in the rooms; on the other hand, non-smokers were satisfied when indoors smoking was banned. Smoking patients also acknowledged that watching the staff smoking was an extra obstacle for them to quit smoking. Non-smoking patients were dissatisfied with the smoking policy of the setting, arguing that it was aimed exclusively at the smokers and raising concerns about their own health (Dickens et al., 2005).

The aim of the present study was to investigate views and attitudes of mental patients regarding smoking.

## 2. Material and Methods

### 2.1 Study Design and Procedure

The study sample consisted of 356 patients, out of 403 initially approached, 142 of them were in-patients and the remaining 214 were staying at rehabilitation units (9 of them within the Hospital area, 34 in the community). The response rate was 88%. Forty-seven patients opted out of the study due to lack of time, low interest in the study, boredom, etc; also there was some difficulty in keeping focused for enough time.

The participants were selected on the basis of several criteria, namely age (>18), citizenship, being smokers, having being diagnosed according to the International Statistical Classification of Diseases and Related Health Problems 10th Revision (ICD-10), and being treated in the Hospital or in an affiliated psychosocial rehabilitation unit.

On the other hand, the exclusion criteria were the following: refusal to sign the informed consent form and/or unwillingness to participate, withdrawal during the interview/questionnaire completion, intellectual disability, autism, or other developmental disorder affecting cognitive functions, current substance abuse and chronic mental disorders combined with severely affected cognitive functions.

### 2.2 Data Collection Process

The patients were approached with great care in order for their autonomy and mental integrity to be safeguarded due to communication difficulties. The research protocol was approved by the Hospital ethics board as well as the National University of Athens ethics board. The study took place from July 2012 until January 2014.

Before the completion of the questionnaire, the participants were informed about the aim of the study and signed the consent form. The participants were free to leave any question unanswered for any reason whatsoever.

The questionnaire administration took place at the place of treatment or stay and lasted for 30-45 minutes. The questionnaires were anonymous and the participants’ rights as laid out in the Declaration of Helsinki, and amended in Tokyo 2004, were strictly observed.

### 2.3 The Study Instrument

The questionnaire “Smoking in psychiatric hospitals - a survey of patients’ views” (Dickens et al., 2005) was chosen for the investigation of views and attitudes, using semi-structured interviews.

The questionnaire items were drawn from existing studies about mentally ill patients who smoke both in-patients and out-patients, and factors affecting smoking cessation. Consequently, the final version of the questionnaire included the following:

- Demographics: Name, age, gender, place of birth, residence, family and marital status, number of children, education level, professional and financial status. Also some data from their medical records were included, such as primary mental disorder, date of first diagnosis, number of previous hospitalizations, legal status, pharmaceutical treatment and compliance, as well as hospitalization department/unit.
- Smoking habit history: Information about smoking habit history of the individuals and if there had been any periods that they had quit. Reasons that encourage or discourage smoking cessation, as well as existing conditions regarding smoking.
- Smokers’ views and attitudes: We investigated views and attitudes of the participants regarding the conditions they lived in and also the relationship with their nurse of reference.
- Smoking and health: The following factors were investigated: Age of smoking initiation, reasons for the initiation, number of cigarettes per day, type of cigarettes, knowledge about the harmful effects of smoking, comorbidity.
- Open question: In the final phase, the participants had the chance to elaborate freely on their own views, beliefs and experiences regarding smoking.

At first the questionnaire was translated from English to Greek (‘forward translation’), and then it was proofread and edited in order for a natural-sounding Greek text to be produced. Then, the Greek text was translated back in English to make sure that no misunderstandings had occurred and to ensure conceptual correspondence between the two texts. After a few corrections, the final version of the questionnaire was ready to be used in the pilot study.

It is noteworthy that this instrument is not a psychometric tool, but a set of questions that assess views and attitudes of the patients. Thus, no standardization or validity/reliability tests are required.

In order to clarify the participants’ views about smoking, smoking cessation and smoking in the Hospital, a Principal Components Analysis took place that included all questions about smoking-related views, not involving any scoring, but solely depended on correlations among them.

The factor analysis interprets 64.8% of the variability of the questions. Using the Kaiser criterion (eigenvalues greater than 1) and varimax rotation, seven distinct factors were selected: “Obstacles in smoking cessation in the hospital settings”; “Views about imposing rules regarding smoking in the hospital settings”; “Views regarding the role of the staff in quitting smoking”; “Quitting smoking due to other health problems”; “Perceived difficulty in quitting smoking”; “Views about smoking habits of persons of reference”; “Motivation for quitting smoking”.

A pilot study took place in order to clarify on the one hand the validity of our research approach (semi-structured interviews based on the questionnaire), and on the other whether this particular questionnaire was easily understood by the patients. More specifically, from March 2012 to June 2012, a hundred patients of the Psychiatric Hospital of Attica were approached, and 80 participated in the study (80% response rate). The pilot study was overall successful and the main study followed (Kourakos & Koukia 2014).

### 2.4 Statistical Analysis

The PASW 18 software was used for data analysis. Statistical significance was set to p= 0.05. The qualitative variables were described based on their absolute (N) and relative frequencies, while quantitative variables were described by mean, standard deviation, median, minimum and maximum values.

A Principal Components Analysis was used to find the factor structure, by employing the correlation coefficients among the variables, and a Varimax orthogonal rotation to interpret the factors.

To investigate the correlation among gender and the type of treatment setting and the participants’ answers, the independent variable was dichotomous (gender or treatment setting) and the dependent variables included all the participants’ answers. In the case of quantitative dependent variables the student’s t-test was used, while when the dependent variables were categorical Pearson’s chi-squared test was applied.

## 3. Results

### 3.1 Demographics and Clinical Characteristics

The overall sample comprised 356 patients, two-thirds of them being men and the rest women. The age ranged between 18 and 84 years, with a mean age of 49.04 years. The majority were born, raised and living in urban areas. Two-thirds of the participants were unmarried, and less than 30% of them had any children. Most of them live with 2 or 3 other family members (ranging from one, being alone, to eight persons). Almost half of the participants were secondary education graduates, and only 15% of them had attended a university. The overwhelming majority had a low income (which at the time of the study was defined as lower than 1000 euros per month); regarding their profession, most of the participants fell under the category of ‘unskilled laborers’ (Table 1).

**Table 1:** Sociodemographic characteristics of the participants

Gender	N (%)
Male	229 (64.3)
Female	127 (35.7)

**Age**	49 (12.2)^α^

**Place of birth and upbringing (up to age 18)**	
Rural	80 (22.5)
Semi-urban	58 (16.3)
Urban	218 (61.2)
**Place of residence**	
Rural	33 (9.3)
Semi-urban	42 (11.8)
Urban	281 (78.9)

**Family status**	

Unmarried	227 (63.8)
Married	41 (11.5)
Widowed	21 (5.9)
Divorced	62 (17.4)
Separated	4 (1.1)
Cohabits	1 (0.3)

**Do they have children?**	

Yes	105 (29.5)
No	251 (70.5)

**Number of family members that live together**	

1	31 (8.7)
2-4	244 (68.6)
>4	81 (22.7)

**Education level**	

No education	16 (4.5)
Primary school	97 (27.2)
(Junior) High school	190 (53.4)
Higher education	53 (14.9)

**Job/Profession**	

Unskilled laborer	107 (30.1)
Skilled laborer	49 (13.8)
Freelancer	39 (11)
Intermediate level office clerk	37 (10.4)
High level office clerk	18 (5.1)
Retired/pensioner	32 (9)
Housework	31 (8.7)
Student	4 (1.1)
None (due to mental disorder)	39 (11)

**Monthly income**	

None	28 (7.9)
Low (up to 999 euros)	299 (84)
Average (1000-2500 euros)	27 (7.6)
High (over 2500 euros)	2 (0.6)

*Note*.

α Mean and standard deviation

Regarding treatment, clinical condition and comorbidity issues: One-third of the participants were under civil commitment; Forty percent were approached while in hospital and the rest in an out-hospital setting (usually a hostel or a boarding house); treatment duration ranged from a few months to 25 years, with a mean duration of 4.4 years. Also, the most common (61.5% of the sample) ICD-10 diagnosis was schizophrenia (F20). 77.8% of the patients fell under the diagnostic codes F20-F29 (Schizophrenia, schizotypal and delusional disorders). Most of the rest (18.2%) fell under the F30-F39 codes (mood [affective] disorders), and only 4% of the participants had another diagnosis, e.g. organic mental disorders, substance use disorder, neurotic disorders, or other. (Table 2)

**Table 2:** Hospitalization data and diagnostic category

Legal status	Ν (%)
Under civil commitment	127 (35.7)
Not under civil commitment	229 (64.3)
**Years of hospitalization**	4,4 (4.8)^α^
**Treatment facility**	
Hospital	142 (39.9)
Guesthouse	102 (28.7)
Boarding house	91 (25.6)
Vocational rehabilitation institutions	21 (5.9)
**Type of treatment setting**	
In-hospital	142 (39.9)
Out-of-the-hospital	214 (60.1)
**Diagnosis (according to ICD-10)**	**Ν (%)**
F01 (Vascular dementia)	1 (0.3)
F07 (Organic personality disorder)	1 (0.3)
F10 (Mental disorders due to alcohol consumption)	2 (0.6)
F11 (Mental disorders due to opioid consumption)	1 (0.3)
F19 (Mental disorders due to multiple substances consumption)	1 (0.3)
F20 (Schizophrenia)	219 (61.5)
F21 (Schizotypal disorder)	9 (2.5)
F22 (Chronic delirium disorders)	7 (2)
F23 (Acute and intermittent psychotic disorders)	7 (2)
F24 (Delirium disorder)	2 (0.6)
F25 (Schizoemotional disorder)	29 (8.1)
F28 (Other non-organic psychotic disorders)	4 (1.1)
F30 (Mania episode)	6 (1.7)
F31 (Bipolar disorder)	30 (8.4)
F32 (Depression episode)	20 (5.6)
F33 (Recurrent depression disorder)	6 (1.7)
F34 (Mood disorders)	3 (0.8)
F40 (Phobic anxiety disorders)	2 (0.6)
F43 (Reaction to intense stress and adjustment disorders)	1 (0.3)
F48 (Other neurotic disorders)	1 (0.3)
F60 (Special personality disorders)	2 (0.6)
F91 (Attitude disorders)	1 (0.3)
F99 (Non defined psychotic disorders)	1 (0.3)

*Note*.

α Mean and standard deviation

Nine out of ten participants were under treatment with neuroleptic drugs. 32.9% of the sample was taking antidepressants, while 36% of them were treated with anti-epileptic drugs. Treatment compliance was by and large good and 77% of the patients had encountered no problems whatsoever. Regarding comorbidity, 38.3% of the participants had also some other medical conditions, namely cardiovascular problems (17.7%), diabetes (12.1%) or respiratory problems (13.2%).

### 3.2 Smoking Habits and Smoking History

Almost all of the participants (97.5%) were regular smokers, who by far (89.5%) smoked manufactured, filtered, cigarettes. On the average, the sample was made up by heavy smokers (a little less than two cigarette packs per day), who had been smoking for about three decades. The average initiation age was under the age of 20 and the main reasons had been: peer pressure (43.9%), curiosity (26.5%), or using smoking as a way to reduce stress (17.5%). The majority of the participants (91%) wanted to quit smoking. Almost two-thirds of them reported previous attempts to quit, and three-fourths of them found some serious obstacles when trying to quit smoking. Also, 62.8% of the participants said they needed help, although it remains unclear whether by help they meant counseling, smoking cessation sessions, or nicotine replacement products. Although most patients were well aware of the fact that smoking is very dangerous, only 40% of them had been advised by a physician to quit smoking promptly for medical reasons.

Regarding questions about smoking in the hospital, 4.8% of the participants said they started smoking during their hospital stay. Nearly all of the patients (96.1%) said that the hospital staff also smoked, mainly in the offices or in the smokers’ lounge. Regarding their persons of reference, 52.5% of the patients said their reference persons also smoked; when asked if they could trust a reference person who smoked, most of the patients said they had no particular opinion about this (47.2%) and 28% of them said they could cooperate better with another smoker.

### 3.3 Views/Attitudes about Smoking Cessation and Hospital Policy Regarding Smoking

Most patients (58.8%) found it very difficult to quit smoking, although they had plenty of information and encouragement (≈90%); they also pointed out that watching the staff smoking didn’t bother them as much as watching other patients smoking (41 % VS 54.8%).

Most patients (67%) agreed with the hospital rules (however they interpreted them), and that the staff should be allowed to smoke (32.4%); but they also wanted more encouragement from the staff to quit or reduce smoking (60.1%), and would like the staff and the visitors to able to smoke with the patients (59%). The patients’ views were not especially concrete or robust but seem to lean towards a more allowing perspective regarding smoking policies.

### 3.4 Correlations

Through a series of univariable analyses, the correlation between gender and all the participants’ answers was investigated.

Both genders showed significant differences regarding marital status (men tend to be unmarried more often compared to women), number of children (women more often have children than men), jobs (men are usually unskilled laborers and women do mostly housework), and income (women do not have any income more often than men who usually have a low or medium income). There were no significant differences regarding education levels, place of birth or place of residence. There were no differences among the two genders regarding age, or number of family members (Table 3).

**Table 3:** Correlation between gender and categorical demographics

Dependent variable		Males Ν (%)	Females Ν (%)	χ^2^	Β.Ε.	P
**Place of birth and upbringing (up to age 18)**	Rural	51 (22.3)	29 (22.8)	0.203	2	0.904
	Semi-urban	36 (15.7)	22 (17.3)			
	Urban	142 (62.0)	76 (59.8)			
**Place of residence**	Rural	25 (10.9)	8 (6.3)	2.801	2	0.246
	Semi-urban	29 (12.7)	13 (10.2)			
	Urban	175 (76.4)	106 (83.5)			
**Family status**	Unmarried	160 (69.9)	67 (52.8)	13.836	5	**0.017**
	Married	24 (10.5)	17 (13.4)			
	Widowed	10 (4.4)	11 (8.7)			
	Divorced	34 (14.8)	28 (22.0)			
	Separated	1 (0.4)	3 (2.4)			
	Cohabits	0 (0.0)	1 (0.8)			
**Has children**	Yes	56 (24.5)	49 (38.6)	7.842	1	**0.005**
	No	173 (75.5)	78 (61.4)			
**Education level**	None	11 (4.8)	5 (3.9)	3.127	3	0.372
	Primary school	56 (24.5)	41 (32.3)			
	(Junior) High school	129 (56.3)	61 (48.0)			
	Higher/highest	33 (14.4)	20 (15.7)			
**Job**	Unskilled laborer	81 (35.4)	26 (20.5)	58.932	8	**0.001**
	Skilled laborer	34 (14.8)	15 (11.8)			
	Freelancer	26 (11.4)	13 (10.2)			
	Intermediate office clerk	22 (9.6)	15 (11.8)			
	Higher office clerk	12 (5.2)	6 (4.7)			
	Pensioner	23 (10.0)	9 (7.1)			
	Housework	1 (0.4)	30 (23.6)			
	Student	3 (1.3)	1 (0.8)			
	None	27 (11.8)	12 (9.4)			
**Income**	None	11(4.8)	17 (13.4)	8.727	3	**0.033**
	Low (up to 999 euros)	198 (86.5)	101 (79.5)			
	Average (1000-2500 euros)	19 (8.3)	8 (6.3)			
	High (>2500 euros)	1 (0.4)	1 (0.8)			

p<0.05.

There were statistically significant differences regarding diagnosis (men suffered more often from schizophrenia, schizotypal or paranoid disorders, while women were more often diagnosed with mood disorders), treatment with neuroleptics (more common with men rather than women) and treatment with antidepressants (more common with women rather than men). No significant differences were found regarding status of civil commitment, department or setting of treatment, treatment with anti-maniac or antiepileptic agents and compliance. No difference was found between years of treatment and gender, and no significant differences were spotted between gender and smoking frequency, type of cigarettes and reasons for smoking initiation (Table 4).

**Table 4:** Correlation between gender and categorical clinical variables

Depended variable		Males Ν (%)	Females Ν (%)	χ^2^	Β.Ε.	P
**Diagnostic category**	Scizophrenia, schizotypal and delusional disorders	193 (84.3)	84 (66.2)	18.162	2	**0.001**
	Emotional/mood disorders	27 (11.8)	38 (29.9)			
	Other	9 (3.9)	5 (3.9)			
**Is the patient under civil commitment?**	Yes	81 (35.4)	46 (36.2)	0.026	1	0.873
	No	148 (64.6)	81 (63.8)			
**Treatment facility**	Hospital	88 (38.4)	54 (42.5)	1.367	3	0.713
	Guesthouse	65 (28.4)	37 (29.1)			
	Boarding house	62 (27.5)	28 (22.0)			
	Vocational rehabilitation units	13 (5.7)	8 (6.3)			
**Type of facility**	In-hospital	88 (38.4)	54 (42.5)	0.570	1	0.450
	Out of the hospital	141 (7.3)	73 (57.5)			
**Treated with neuroleptics**	Yes	212 (92.6)	107 (84.3)	6.079	1	**0.014**
	No	17 (7.4)	20 (15.7)			
**Treated with antidepressants**	Yes	60 (26.2)	57 (44.9)	12.921	1	**0.001**
	No	169 (73.8)	70 (55.1)			
**Treated with antimanics**	Yes	9 (3.9)	10 (7.9)	2.515	1	0.113
	No	220 (96.1)	117 (92.1)			
**Treated with anti-epileptics**	Yes	82 (35.8)	46 (36.2)	0.006	1	0.938
	No	147 (64.2)	81 (63.8)			
**Compliance**	Full	180 (78.6)	94 (74.0)	2.133	3	0.545
	Intermittent	22 (9.6)	18 (14.2)			
	Problematic	25 (10.9)	13 (10.2)			
	Stopped treatment without medical advice	2 (0.9)	2 (1.6)			

p<0.05

Men were found to smoke a significantly larger number of cigarettes per day compared to women (36.70 vs 30.82, p=0.002). They had also started smoking at a younger age than women (18.42 vs 19.89, p=0.077), and had been smoking for more years than women (30.64 vs 28.34, p=0.108), but these differences were not statistically significant. Women asserted more often their intention to quit smoking compared to men (96.1 vs 88.1, p=0.012). No significant differences were found among men and women regarding previous attempts to quit, obstacles to quit, help to quit, medical advice to quit and knowledge about the hazards of smoking. Also, no significant differences were found among men and women regarding smoking in the hospital, the smoking habits of their reference persons and their own attitudes about quitting smoking.

Univariable analyses were also carried out in order to establish correlations between the type of the treatment setting (in-hospital or in the community) and the participants’ answers. Statistically significant differences were found regarding marital status (p=0.001), in-patients being more often married (33.2%) and out-patients unmarried (68.2%). Regarding education level, in-patients were more often high school graduates (56.3%), while out-patients were more often primary level graduates (29.4%). Profession-wise, out-patients were more often unskilled laborers (36.4%), and in-patients were pensioners (12%); regarding income, out-patients had lower income levels than in-patients (90.2% vs 74.6%). No significant differences were found regarding place of birth or residence and if they had any children or not. It was found that patients treated in out-hospital settings (out-patients) were significantly older (50.81vs 46.36, p=0.001) and lived with several other family members, compared to in-patients (3.82 vs1.264, p=0.003).

Also, in-patients were more often under civil commitment (61.3% vs 18.7%, p=0.001) and were administered more often anti-epileptics (42.3% vs 31.8%, p=0.044), while on the other hand, out-patients showed better compliance compared to in-patients (85% vs 64.8%, p=0.001). No significant differences were found regarding diagnosis and treatment with antidepressants or anti-maniac drugs, while in-patients had in general more years under treatment than out-patients (5.75 vs 3.55, p=0.001).

Regarding the patients’ smoking habits in relation to the treatment setting, in-patients smoke more cigarettes per day (37.03 vs 32.98, p=0.027). As regards smoking initiation age, out-patients started smoking at an older age (19.65 vs 17.89, p=0.030), and in-patients thought more often that they started smoking because of peer pressure (59.2%), while out-patients attributed starting smoking to intense stress and personal problems (22.5%). Out-patients thought they had been smoking for more years than in-patients, yet this difference was not statistically significant (30.83 vs 28.30, p=0.072). Also, no significant differences were found regarding smoking frequency and type of cigarettes.

There was a significant correlation between the type of the treatment setting and the patients’ answers about smoking cessation. More specifically, hospital in-patients showed a greater intention to quit smoking compared to out-of-hospital patients, who in turn reported more previous attempts to quit smoking. As regards obstacles in quitting smoking, out-of-hospital patients reported more often than in-patients finding obstacles. As far as smoking hazards were concerned, in-patients gave more often the answer “None”, while out-patients gave more often the answer “Low hazard”. No significant differences were found regarding whether they needed help to quit smoking or if a physician had advised them to quit smoking (Table 5).

**Table 5:** Correlation between type of treatment facility and smoking habits within the facility

Depended variable		In-hospital. Ν (%)	Out-hospital. Ν (%)	χ^2^	Β.Ε.	P
**Intention to quit smoking**	Yes	134 (95.0)	188 (88.3)	4.733	1	**0.030**
	No	7 (5.0)	25 (11.7)			
**Previous attempts to quit smoking**	Yes	76 (56.7)	143 (75.7)	12.891	1	**0.001**
	No	58 (43.3)	46 (24.3)			
**Obstacles to quit smoking**	Yes	22 (16.4)	57 (30.2)	8.013	1	**0.005**
	No	112 (83.6)	132 (69.8)			
**Would need help to quit?**	Yes	58 (65.7)	115 (60.8)	0.782	1	0.377
	No	46 (34.3)	74 (39.2)			
**Medical advice to quit smoking promptly**	Yes	58 (40.8)	77 (36.3)	0.738	1	0.390
	None	84 (59.2)	135 (63.7)			
**Perception about hazards of smoking**	High	88 (62.0)	133 (62.4)	12.446	2	**0.002**
	Low	17 (12.0)	50 (23.5)			
	None	37 (26.1)	30 (14.1)			
**Smoking initiation in the hospital**	Yes	4 (2.8)	13 (6.1)	1.980	1	0.159
	No	137 (97.2)	200 (93.9)			
**Does the staff smoke?**	Yes	138 (97.2)	204 (95.3)	0.778	1	0.378
	No	4 (2.8)	10 (4.7)			
**Place where staff smokes**	Smoking lounge	45 (32.6)	35 (17.2)	11.393	2	**0.003**
	Offices	81 (58.7)	142 (69.6)			
	Outside	12 (8.7)	27 (13.2)			

p<0.05

A significant correlation was found between the type of the treatment setting and the patients’ answers about where the staff used to smoke. In-patients’ answer was more often the “smoking lounge”, while out-patients’ most common answer was “outside”. No differences were found regarding if the staff smoked and if the patients took up smoking in the hospital.

When it came to persons of reference, all out-patients knew their reference person (100%), compared to 89.4% of the in-patients; also, most out-patients were not aware if their reference person smoked (44.4%), while most in-patients (60.3%) admitted that their reference persons smoked. No significant differences were found for the questions “I would trust more a person of reference that does not smoke compared to one who smokes” and “I can cooperate better with a person of reference that smokes than a non-smoker”.

A statistically significant correlation was found between attitudes towards smoking cessation and type of treatment setting. More specifically the following questions showed significant differences: “I don’t have enough information to quit smoking”, “A smoke-packed room would deter me from quitting smoking”, “Watching other patients smoking would discourage me from quitting smoking”, “Watching the staff smoking would make it difficult for me to quit smoking”; most out-patients agreed with the above statements, and consequently they see more obstacles in quitting smoking than in-patients. No significant differences were found regarding the statement “There is not enough encouragement from the staff to quit smoking”. When it came to the statement “It’s too hard for me to quit smoking”, in-patients agreed more but the difference was not statistically significant. There was also a significant correlation between type of treatment setting and the patients’ positions on the statements “The setting’s rules on smoking are right on target” and “The staff should encourage smokers to quit or reduce smoking”, where out-patients agreed more than in-patients. The rest statements regarding hospital policy on smoking did not yield any significant differences (Table 6).

**Table 6:** Correlation between type of treatment setting and the patients’ views on smoking cessation and the hospital’s smoking policy

Depended variable	In-hospital. (Ν=134)		Out-hospital. (Ν=189)		T	B.E.	P
	Mean	SD	Mean	SD			
**There is not enough information for me to quit smoking**	1.77	0.587	1.98	0.815	-2.616	321	**0.009**
**There is not enough encouragement from the staff to quit smoking**	2.54	0.898	2.53	0.965	0.078	321	0.938
**A smoke-packed room would make it hard for me to quit smoking**	2.87	1.120	3.19	1.109	-2.524	321	**0.012**
**Watching other patients smoke would make it hard for me to quit smoking**	2.94	1.155	3.33	1.100	-3.056	321	**0.002**
**Watching the staff smoke would make it hard for me to quit smoking**	2.72	1.108	3.01	1.123	-2.330	321	**0.020**
**It is very difficult for me to quit smoking**	4.05	0.904	3.87	0.970	1.683	321	0.093
**The staff should be allowed to smoke when at work**	3.37	0.993	3.22	1.022	1.340	354	0.818
**Staff should be allowed to smoke with the patients^1^**	3.39	0.905	3.53	0.903	-1.026	186	0.306
**Visitors should be allowed to smoke with the patients**	3.28	1.006	3.31	1.006	-0.245	354	0.806
**The facility’s smoking rules are on target**	3.21	0.866	3.79	0.719	-6.790	354	**0.001**
**The staff should encourage smokers to reduce or quit smoking**	3.15	0.967	3.69	0.919	-5.305	354	**0.001**
**The staff should make an example and avoid smoking**	2.94	0.991	3.12	1.026	-1.645	354	0.101

p<0.05;

1 In this item, 84 patients from in-hospital units and 104 patients from out-of-hospital facilities gave an answer.

## 4. Discussion

The sample comprised smoking patients with a variety of mental disorders from a major hospital in Athens. By analyzing places of birth and residence, it was shown that the sample came from all types of regions of Greece, urban, semi-urban and rural.

The sample size was deemed satisfactory, not only because of the number of the participants, but also because of the sampling method. All of the (hospital and out-of-hospital) patients were individually approached and strict selection and exclusion criteria were used.

Regarding the socio-demographic characteristics of the participants, they were for the most part unmarried, childless males, high school graduates, of low income, professionally unskilled, who lived mainly in urban areas with 2-4 other family members.

Taking their clinical characteristics into account, most patients suffered from schizophrenia (61.5%), while other diagnoses such as schizo-emotional disorder, bipolar disorder and depression were also common. A significant chunk of the sample (35.7%) were under civil commitment, while there was much variety regarding their treatment settings, since a large proportion had been treated in the hospital (39.9%) and the rest had been treated in out-of-hospital institutions, namely guesthouses (28.7%), boarding houses (25.6%) and professional rehabilitation institutions (5.9%). Also, almost all of them were under treatment with neuroleptics (89.6%), and several of them were taking antidepressants (32.9%) and anti-epileptics (36%). Treatment compliance was very good and cardiovascular conditions were the most common co-existing medical problems (17.7%).

All participants were smokers because of the purpose of the present study, thus we are not able to assess all of the patients’ smoking frequency in general. Nevertheless, it was empirically observed that smoking was pretty common in this population, a finding internationally acknowledged. Since the sample of the present study was made up by patients with schizophrenia or schizo-emotional disorder, it can be compared with studies with similar samples. More specifically, in the United States, the National Comorbidity Survey showed that 41% of mental patients were smokers (Lasser et al., 2000). Psychotic and bipolar patients tended to smoke in greater numbers (67.9% and 82.5%, respectively). Again in the U.S., the National Health Interview Survey (2007) found that 59.1% of patients living in the community had schizophrenia (McCave, McKnight-Eily, Davis, & Dube, 2010). A relevant 2001 review concluded that almost 60% of mental patients are smokers. According to a study that took place in Colorado, U.S.A., 39% of mental patients who had received treatment from the public healthcare providers were smokers, and the most common diagnoses were schizophrenia, schizo-emotional disorder and bipolar disorder (Morris et al., 2006). Another study in Minnesota, USA, (Lineberry, Allen, Nash, & Galardy, 2009) found that 45.9% of mental patients were smokers, while in patients with psychotic disorders the percentage went up to 77.1%. It is widely accepted that smoking rates are higher in in-patients (81.5%) than out-patients (68.4%) (Kalman, Morissett, & George, 2005).

Males made up the majority of our sample (almost 2:1). As it was also found in the study about schizophrenic patients living in the community (Hou et al., 2011), there was a correlation between smoking and male gender. Other authors have found similar results (Salokangas, Honkonen, Stengard, Koivisto, & Hietala, 2006). In general there is a significant correlation between smoking and schizophrenia, on the one hand, and male gander on the other (De Leon & Diaz 2005). Almost all of the participants (97.5%) smoked regularly everyday. Accordingly, a similar study (McCave et al., 2007) found that 85.7% of smoking schizophrenic patients smoke daily compared to 77.1% of the smokers that belong to the general population. Moreover, 89.5% smoked manufactured cigarettes, and so does the general population in Greece (European Commission, TNS opinion and social: Tobacco, 2010). In our sample, the average smoking initiation age was 19 years. In Scotland (Kelly & McCreadie, 1999) the general initiation age was 17 years. A study in Australia (Baker et al., 2007) showed that smoking initiation age for mental patients was around 18 years.

Our sample consisted of heavy smokers that smoked 34.6 cigarettes per day. According to the National Comorbidity Survey (Lasser et al., 2000), in the United States the average cigarette consumption was 26.2 cigarettes per day, but that figure applied to all the patients who lived in the community no matter what their diagnosis was. The previously mentioned study in Scotland (Kelly & McCreadie, 1999) showed that 68% of the patients smoked 25 or more cigarettes daily. A study in Australia (Baker et al., 2007) found that psychotic patients smoked about 30 cigarettes daily. The number of cigarettes consumed daily by patients should be assessed compared to the total number of cigarettes consumed daily by the general population, which in Greece and Cyprus is 21 cigarettes or more-the highest in the European Union (European Commission, TNS opinion and social: Tobacco, 2010).

As previously mentioned, almost the entirety of the sample was under treatment with neuroleptics. It has been assumed that many schizophrenic patients resort to smoking in order to control some side effects of the treatment (Ziedonis et al., 2008). It has been found that the administration of haloperidol, the most common antipsychotic medication, can increase smoking frequency in mental and non-mental patients (Levin, Wilson, Rose, & McEvoy, 1996) while nicotine alleviates some physical side effects, such as haloperidol-induced akathisia (Barr, Procyshyn, Hui, Johnson, & Honer, 2008). Moreover, it has been found that neuroleptics such as haloperidol can affect negatively spatial memory and speed of information processing, and both of those symptoms can be reduced or even reversed with nicotine (Levin et al., 1996). Finally, it has been argued that neuroleptic drugs increase the pleasure caused by smoking both as a habit and as a psychomotor activity (Barr et al., 2008).

In that particular study, 91% of the patients intented to quit smoking. The initial study that inspired our study has found a similarly high percentage (97.1%) (Dickens et al., 2005). A study that took place in the city of Buffalo, N.Y., (Carosella, Ossip-Klein, & Owens, 1999) found that 33% of the patients had no intention of quitting smoking whatsoever, 40% expressed their will to quit smoking within the next six months, and 23% of them intended to do so in the next month.

In our sample only 67.8% of the participants had already attempted at least once to quit smoking. In a study by Dickens et al. (2005) the respective percentage was 97.1%. In a study in Australia (Baker et al., 2007), it was found that most psychotic patients had already 2-3 failed quit attempts. Overall, successful smoking cessation in schizophrenic patients is lower than the general population: A combination of studies that took place in five countries (De Leon, & Diaz, 2005) showed that 9% of the patients succeed in quitting smoking compared to 14%-49% of the general population.

Despite being heavy smokers, most participants acknowledged that smoking was dangerous (62.3%). Similarly a study in the USA (Carosella et al., 1999) found that 68% of the participants agreed that smoking was harmful to their health, while in Liverpool (Smith & O’Callaghan, 2008) 92.6% of the patients thought that smoking was a dangerous habit. On the other hand, 18.9% of the participants said smoking wasn’t dangerous at all. Similarly, a study in India (Pattanayak, Sagar, & Jain, 2012) found that patients were not aware of the smoking-related range of medical problems, did not pay any attention to the warnings printed on the cigarette packs and thought that they had less chance of developing cancer compared to other smokers of similar age.

Moreover, 75.5% of our sample thought there were no significant obstacles for them to quit smoking. Another study in Chicago, Illinois (Spring, Pingitore, & McChargue, 2003), found that although schizophrenic or depressive patients knew clearly the draw backs of smoking, nevertheless they found it too pleasant and rewarding, so they thought a major reward was needed for them to quit smoking. Other studies regarding the patients’ opinions also found that many of them actually enjoy smoking (Barr et al., 2008), they feel that smoking alleviates their stress and stimulates them (Baker et al., 2007), that it is central to their lives and helps them cope with depression and anxiety (Snyder, McDevitt, & Painter, 2008), or that it relieves them from boredom and loneliness (Ziedonis, & Williams, 2003).

Similarly with a study in Great Britain (Ratschen, Britton, Doody, & McNeill, 2010), the majority of our participants (62.8%) felt they needed help to quit smoking. The rest said they prefered to quit smoking on their own, and the same said 80.8% of the participants in a study in India (Pattanayak et al., 2012). Regarding type of help, nicotine replacement products were not popular with our sample (a mere 25.3%), and the same went for smoking cessation groups (only 25.8%). Support and counseling was much more prefered (42.8%). In the initial study (Dickens et al., 2005) the respective figures were 70.6%, 32.4% and 17.6%. The comparison between those figures shows that Greek patients are not accustomed neither to nicotine replacement therapies (which are exempt from insurance coverage) nor to smoking cessation groups (that are by and large rare in Greece). It seems that this situation stands also for the general smoking population, since apparently less than 3% of smoking persons use some kind of nicotine replacement therapy (European Commission, TNS opinion and social: Tobacco, 2010).

Only 31.8% of our participants had been advised by their physician to quit smoking on medical grounds. In a similar study (Pattanayak et al., 2012), 15.4% of those who intended to quit smoking had been advised by a physician to do so (and they made up 52% of the sample and 29% of the total sample).

Regarding several smoking cessation questions, our participants agreed with the sample of the study conducted by Dickens et al. (2005). For instance, 84.2% agreed that “it is too hard for me to quit smoking” (compared to 73.5% in the other study (Dickens et al., 2005), while 21.7% said “there is not enough encouragement from the staff for me to quit smoking” (compared to 29.4%). For the most part, our participants gave similar answers with the participants in Dickens et al. (2005) albeit less strongly worded. For instance, the statement “A smoke-packed room will make it difficult for me to quit smoking” was accepted by 48.3% of our participants, compared to 58.8% in the other study (Dickens et al., 2005). “Watching the staff smoking will make it difficult for me to quit smoking” was the only statement where there was a divergence, since 52.7% of our participants disagreed, compared to 55.9% of Dickens’ participants who agreed (Dickens et al., 2005).

Regarding the participants’ views on the hospital’s smoking policy, the majority thought it should be allowed for staff and visitors to smoke with the patients (60.5% and 60.0% of the answers respectively), although Dickens’ findings were a little higher (77.8% and 82.2% respectively) (Dickens et al., 2005). Similarly, 66.9% of the patients agreed with the statement “Smoking rules are to the point” and 60.1% agreed with the statement “The staff should encourage smokers to reduce or quit smoking”, compared to Dickens’ 88.2% and 55.6% respectively (Dickens et al., 2005). For the last statement “The staff should set an example and avoid smoking in front of the patients” our participants were rather divided (39.3% agreed and 36.6% disagreed), while 26.7% of Dickens’ participants agreed and 53.3% disagreed (Dickens et al., 2005).

In our study, the patients’ answers about the workers’ smoking habits were relatively consistent. More specifically, 52.7% of our participants disagreed with the statement “Watching the workers smoke will make it hard for me to quit smoking”, and 52.3% of them agreed with the statement “It should be allowed for the staff to smoke within the hospital settings”, while in the original study (Dickens et al., 2005) the respective findings were inconclusive. On the other hand the majority of our participants’ answers did not show extreme opposition, remaining more or less neutral.

The participants’ answers were grouped together in conceptually homogeneous categories by applying a factor analysis (principal factor method). Interestingly, the patients seem to differentiate their views about hospital rules from what they feel can help or deter them from quitting smoking. Also, apparently most participants view quitting smoking because of health problems and the smoking habits of reference persons as two distinct cases compared to similar information about other staff members.

The participants’ attitudes regarding smoking cessation, smoking indoors and possible attempts to quit in the future, were affected by various factors. Socio-demographic factors such as gender, existence of children, education and income, along with other factors such as number of previous hospitalizations, treatment compliance, and diagnosis, duration of smoking and age of initiation were correlated in univariable analyses with one or more of the factors that resulted from the principal factor analysis.

The most significant factors were the following: type of treatment setting (in-hospital or out-of-the-hospital), legal status (civil commitment), depression (as a diagnosis or indirectly because the patient received antidepressants), and age.

Gender and type of treatment setting were correlated with the questionnaire items. Gender was correlated with demographics (family status, job, income, and children) and clinical characteristics (diagnosis, treatment with neuroleptics or antidepressants). The interaction between demographics and clinical characteristics on the one hand and the smokers’ sex was also identified in a study in Vancouver, Canada (Johnson et al., 2010), where a different tobacco use profile was found between males and females. Also, in our study men were found to be heavier smokers than women and less motivated to quit smoking. No statistically significant differences were spotted in relation to their views on smoking cessation and smoking within the hospital settings. Findings such as men being heavier smokers and being smokers for more years have been acknowledged by other authors too (Langenecker et al., 2009). Nevertheless, in our study men were less determined to quit smoking. Type of treatment setting was correlated with several questions: demographics (family status, education level, job, income, age, number of other family members), clinical characteristics (civil commitment, treatment with anti-epileptics, treatment compliance, and years of treatment), psychopathology and functionality (as assessed by the questionnaires) and also smoking-related attitudes. In-patients were found to be heavier smokers, were more determined to quit smoking, and thought smoking was less dangerous compared to out-patients. Overall, out-patients felt there were more obstacles for them to quit smoking, thought that the staff should encourage them to quit or reduce smoking and agreed with the smoking rules of their setting.

In general, the present study is, as far as we know, the first of its kind to study systematically the mental patients’ attitudes regarding smoking and smoking policies in different treatment settings, by using such a large sample and employing the patients’ own notions and ideas and not those of the authors. It would be interesting if the study could be cross-sectional between various healthcare facilities in the future.

## 5. Conclusions

Total sample consisted of patients under treatment in a major mental hospital in Athens and its affiliated out-of-the-hospital settings. It is possible that other patients who are being treated in psychiatric departments of general hospitals or other primary healthcare settings may differ regarding their demographics/clinical characteristics and their views about smoking and smoking cessation.

As a conclusion, although almost all mental patients smoke, they seem to be receptive to quitting smoking, since two thirds of them have already tried to quit, but one-third of the patients find smoking a little or not at all dangerous. They more or less accept smoking in the hospital settings and are not against the staff smoking, and seem to resist any attempts to change this situation. It is important that the findings of this study to be taken into consideration in any systematic attempts to constrain smoking in mental health facilities.
